# Identification of a Selective PDE4B Inhibitor From *Bryophyllum pinnatum* by Target Fishing Study and *In Vitro* Evaluation of Quercetin 3-*O-α-L*-Arabinopyranosyl-(1→2)-*O-α-L-*Rhamnopyranoside

**DOI:** 10.3389/fphar.2019.01582

**Published:** 2020-01-22

**Authors:** Estela M. G. Lourenço, Júlia M. Fernandes, Vinícius de F. Carvalho, Raphael Grougnet, Marco A. Martins, Alessandro K. Jordão, Silvana M. Zucolotto, Euzébio G. Barbosa

**Affiliations:** ^1^ Laboratório de Química Farmacêutica Computacional, Departamento de Farmácia, Universidade Federal do Rio Grande do Norte, Natal, Brazil; ^2^ Laboratório de Produtos Naturais Bioativos, Departamento de Farmácia, Universidade Federal do Rio Grande do Norte, Natal, Brazil; ^3^ Laboratório de Inflamação, Fundação Oswaldo Cruz, Rio de Janeiro, Brazil; ^4^ Laboratoire de Pharmacognosie, Faculté de Pharmacie, Université Paris Descartes, Paris, France

**Keywords:** *Bryophyllum pinnatum*, natural products, PDE4, Flavonoid, inverse virtual screening, molecular dynamics

## Abstract

Natural products are considered an important source of bioactive compounds especially in biodiversity-rich countries like Brazil. The identification of potential targets is crucial to the development of drugs from natural sources. In this context, *in silico* methodologies, such as inverse virtual screening (target fishing), are interesting tools as they are a rational and direct method that reduces costs and experimental time. Among the species of Brazilian biomes, *Bryophyllum pinnatum* (Lam.) Oken, native to Madagascar, is widely used by the population to treat inflammation conditions. It has a remarkable presence of flavonoids, including quercetin 3-*O-α-L*-arabinopyranosyl-(1→2)-*O-α-L-*rhamnopyranoside (**1**), considered one of its major compounds. However, until now there were no studies addressing its putative mechanism of action and explaining its pharmacological action. The enzyme PDE4B, known as an antiinflammatory protein, was indicated as a promising target by target fishing methods. This activity was confirmed by *in vitro* enzymatic inhibition, and an expressive selectivity of PDE4B over PDE4A was demonstrated. The interactions were investigated through molecular dynamics simulations. The results were pioneering, representing an advance in the investigation of the antiinflammatory action of *B. pinnatum* and confirm the potential of the flavonoid as a chemical extract marker. Also, the flavonoid was shown to be a promising lead for the design of other selective PDE4B blockers to treat inflammatory diseases.

## Introduction

Historically, natural products and their derivatives have a remarkable importance in the process of discovery and development of new drugs, being responsible for several of the recently approved new chemical entities (Patridge et al., 2016) and for about 50% of all drugs approved in clinical use (Horbal et al., 2018). The identification of the mechanism of action for these natural products may be a great tool for modern drug design (Xu et al., 2018). However, the search for targets still follows the traditional methods with the execution of direct biological analyses, contributing to the known challenges that involve the process of drug discovery. In this sense, the utilization of *in silico* techniques has been increasing over the last decades (Freires et al., 2017). These methods are considered rational, reduce costs, experimental time, and number of biological models. It has been important in target identification and discovery of novel potential drugs (Wadood et al., 2013).

Among the *in silico* methodologies, virtual screening (VS) represents one of the biggest advances in drug design (Rodrigues et al., 2012). This method had become a primary component in drug development, often combined with homology modeling, QSAR studies, molecular dynamics simulations (Pérez et al., 2016) and *in vitro* assays (Espargaró et al., 2017). The VS approach, divided into structure and ligand-based, uses large libraries with the objective to identify a compound that is capable of binding to a molecular target, generally a protein or enzyme (Rester, 2008). There are several successful studies that have utilized VS to identify promising ligands and targets, including researches involving secondary metabolites (Xu et al., 2018). Among its advantages, the application of this tool in the target identification of natural products can reduce the number of biological assays and consequently, the amount of isolated compounds used in this process.

**Figure 1:**
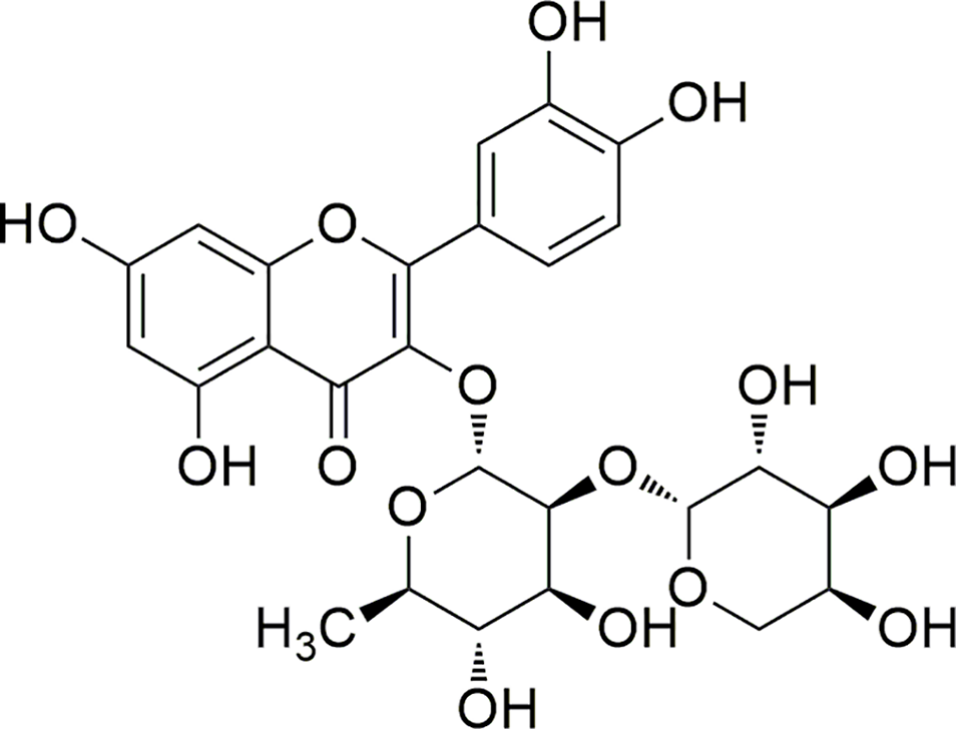
Structure of compound 1.

The Brazilian biomes are considered a large source of bioactive compounds, hosting 15%–20% of all global biodiversity (Barreiro and Bolzani, 2009). Among the plants of the Brazilian biomes, *Bryophyllum pinnatum* (Lam.) Oken (*B. pinnatum*), native to Madagascar, is important in the therapeutic context since it is widely used to treat inflammation conditions (Panyaphu et al., 2011; Ezuruike and Prieto, 2014; Sreekeesoon and Mahomoodally, 2014). Botanic synonyms as *Bryophyllum calycinum* and *Kalanchoe pinnata* can be found (Fernandes et al., 2019). This species is rich in phenolic compounds, particularly flavonoids (Nascimento et al., 2015). One of its major compounds is a glycosylated quercetin derivative identified as quercetin 3-*O-α-L*-arabinopyranosyl-(1→2)-*O-α-L-*rhamnopyranoside (**1**) (**Figure 1**) (Muzitano et al., 2006; Fernandes et al., 2016).

This compound, first derivative isolated from *B. pinnatum*, was found in higher proportions than the other secondary metabolites by HPLC analysis of the extract. Interestingly, previous studies had shown that the increase of compound **1,** caused by the different conditions, can be related to the improvement of the effects, as antioxidant properties (Nascimento et al., 2013). Other activities of compound **1** were also been discussed (Muzitano et al., 2006; Sobreira et al., 2017). In addition, the 3-*O* diglycosidic bond and the dimer rhamnose–arabinose are not common structure features and this flavonoid was described for few other species from different botanical families, however not as their major compound (Fernandes et al., 2019). Therefore, the compound **1** may be considered a promising chemical marker of the extract of *B. pinnatum,* making it also important to the conduction of deeper studies aiming to investigate its biological activities (dos Santos Nascimento et al., 2018). However, it is unknown the mechanism of action and/or some probable targets. In addition, this information represents a crucial factor in the discovery and development of new bioactive compounds based on its structure (Xu et al., 2018).

Thus, the aim of this study was to investigate the potential mechanism of action for compound **1** using *in silico* methodologies, known as target fishing (TF), and molecular dynamics simulations, combined with *in vitro* enzymatic inhibition. This investigation can assist in the selection of compound **1** as a possible chemical extract marker and also to the discovery of this flavonoid as a potential lead compound.

## Materials and Methods

### Plant Material

The leaves of *B. pinnatum* were collected in November 2016 from the Escola Agrícola de Jundiaí (EAJ-UFRN), Macaíba city, Rio Grande do Norte state, Brazil, at the following coordinates: 5° 51’ 30” S and 5° 51’ 30” W. A vouch specimen (UFC. n° 57335) was deposited at the Herbario Prisco Bezerra (EAC) after its botanic identification by Dr. Rúbia Santos Fonseca. The plant material collection was made under the approval of Brazilian Authorization and Biodiversity Information System (SISBIO), process number 35017. Furthermore, the research was registered in the National System of Management of the Genetic Patrimony and Associated Traditional Knowledge-SISGEN under number A7EA798.

### Extraction and Isolation of Compound 1

The fresh leaves without stems (1.6 kg) were subjected to a turbo extraction for 5 min in an industrial blender, using EtOH: H_2_O (1:1, v/v) at a plant: solvent proportion of 1:1 (w/v). Subsequently, the hydroethanolic extract (HE) was filtered and the volume was concentrated by rotaevaporation (model v-700, Buchi^®^). With the HE, a liquid-liquid extraction was made with three solvents of different degrees of polarity. Through this process, the dichloromethane (CH_2_Cl_2_), ethyl acetate (AcOEt), and *n*-butanol (*n*-BuOH) fractions were obtained.

For the isolation of compound **1**, the *n*-BuOH fraction (2 g) was selected and submitted into a column chromatography (silica gel 60) with gradient elution (30%–0% CH_2_Cl_2_: 70%–100% AcOEt and 95%–0% AcOEt: 5%–100% MeOH) resulting in nine fractions (G1–G9). The fractions G3 (520.7 mg) and G4 (372.5 mg) were purified separately through a process of size-exclusion column chromatography using Sephadex LH-20 as stationary phase and MeOH as solvent. The process of purification ended with five subfractions (F1–F5) for both primary fractions, G3 and G4. Between them, the subfractions F4 (from G3) and F5 (from G4) showed the same chemical profile by TLC, characterized by the presence of just one yellow band. These subfractions were reunited and submitted to a final purification process through preparative high-performance liquid chromatography (HPLC-UV) (Shimadzu^®^) using an isocratic elution (80% Milli-Q water and 20% Acetonitrile), a semipreparative column C18 Luna Phenomenex^®^ (250 mm × 10 mm, 5 µm), flow of 4 ml/min and analysis time of 25 min. All fractions obtained during the entire purification process, were monitored by thin layer chromatography (TLC) (Merk silica gel 60) and the bands were visualized by UV (254 and 365 nm). After the purification, the isolation of compound **1** (34.8 mg) was confirmed by UPLC-DAD system used was a Shimadzu Model LC-20AD, with DAD detector SPD-M20A and software LabSolutions. Then, a Phenomenex Kinetex RP-18 column (150 mm × 4.6 mm, 2.6 μm particle size) equipped with a Phenomenex security guard column (4.0 mm × 2.0 mm i.d.) was used. The eluents were: (A) trifluoroacetic acid (TFA) 0.3% and (B) acetonitrile. The following gradient (v/v) was applied: 7%–15% B, 0–3 min; 15%–20% B, 3–12 min; 20%–22% B, 12–30 min; 30 min total analysis time. Flow elution was 0.7 ml/min, and 12 μl of each sample was injected. The UV-DAD detector was programmed to wavelength 200–500 nm and the chromatograms were plotted at 254 and 340 nm. The samples were resuspended in methanol: water, 1:1 (v/v) and the final concentration was 2 mg/ml for the extracts. HPLC-grade acetonitrile and trifluoracetic acid (TFA) were provided from J. T. Baker (Brazil). Water was purified with a Milli-Q system (Millipore). Samples and solvents were filtrated through a membrane (pore-size 0.45 μm) and degassed. The analyses were performed in triplicate. The UPLC-DAD analysis confirmed a purity percentage of approximately 93% (**Figure S1**, **Supplementary Material**). The detailed conditions of all column chromatography are described in **Table S1**, in **Supplementary Material**.

### Structural Characterization of Compound 1

**Quercetin 3-*O-α-L*-arabinopyranosyl-(1→2)-*O-α-L-*rhamnopyranoside (1):** amorphous yellow solid; ^1^H NMR (DMSO-*d*_6_, 500 MHz) *δ* 7.32 (1H, s, H-2’), 7.25 (1H, d, *J* = 8.30 Hz, H-6’), 6.87 (1H, d, *J* = 8.25 Hz, H-5’), 6.38 (1H, s, H-8), 6.18 (1H, s, H-6), 5.29 (1H, s, H-1’’), 4.08 (1H, d, *J* = 7.15 Hz, H-1’’’), 4.00 (1H, s, H-2’’), 3.21 (1H, d, *J* = 12.0 Hz, H-5’’’), 3.12 (1H, t, *J* = 9.50 Hz, H-2’’’), 3.12 (1H, t, *J* = 9.50 Hz, H-3’’’), 0.90 (1H, d, *J* = 6.10 Hz, H-6’’); ^13^C NMR (DMSO-*d*_6_, 125 MHz) δ 177.92 (C, C-4), 164.48 (C, C-7), 161.40 (C, C-9), 157.14 (C, C-5), 156.56 (C, C-2), 148.78 (C, C-4’), 145.38 (C, C-3’), 134.44 (C, C-3), 121.03 (CH, C-6’), 120.61 (C, C-1’), 115.73 (CH, C-2’), 115.54 (CH, C-5’), 106.5 (CH, C-1’’’), 104.2 (C, C-10), 101.05 (CH, C-1’’), 98.88 (CH, C-6), 93.84 (CH, C-8), 80.68 (CH, C-2’’), 72.56 (CH, C-4’’), 71.88 (CH, C-3’’’), 71.14 (CH, C-2’’’), 70.41 (CH, C-3’’), 70.41 (CH, C-5’’), 67.90 (CH, C-4’’’), 65.93 (CH_2_, C-5’’’), 17.56 (CH_3_, C-6’’’).

The NMR spectra are described in **Supplementary Material**.

### TF Strategies

The 3D structure model of compound **1** was built using the program MarvinSketch 16.9.5 (ChemAxon Ltd.). The program AutoDockVINA (Olson and Trott, 2010) was used to obtain the required files for the inversed VS search. The target library used for structure-based inverse VS was constructed from the RCSB PDB Protein Data Bank (Bernstein et al., 1978) and was comprised of more than 9,000 structures with bound ligands. The TF was performed by molecular docking simulations using AutoDockVINA (Olson and Trott, 2010) automated *ad hoc* by shell scripting. The grid box used for the search was big enough to contain roughly the binding site of the targets and the original ligands were deleted before each simulation. The lowest free energy from the empirical score was used to rank the results. The best 25 results of each compound were selected for manual evaluation.

For the search of targets for compound **1**, it was necessary to screen a simplified moiety (quercetin) as ligand due to the exaggerated number of rotatable bonds present on this flavonoid. The best 50 results were selected for visual evaluation to ensure if the size of the active site of the targets was sufficiently large to accommodate the glycosides bound to compound **1** as a whole. The most promising targets were then selected for the docking with the complete structure of the glycosylated flavonoid.

The compound **1** was also submitted to a ligand-based VS using the web tool SwissSimilarity (Zoete et al., 2016), developed by SIBS Swiss Institute of Bioinformatics, to make a rapid ligand-based VS of small to unprecedented ultralarge libraries of small molecules, including the screening of drugs and bioactive and commercial molecules. With this web tool, the prediction can be carried out using six different approaches, such as 2D molecular fingerprints and super positional and fast nonsuper positional 3D similarity molecules (Zoete et al., 2016). In this study, the combined method was selected for screening and the library of bioactive ligands was used for comparison with the isolated compound constructed from the RCSB PDB Protein Data Bank server (Bernstein et al., 1978). The results were filtered with the aim to consider only similarity coefficients above 0.500 for each ligand of this work.

### PDE4 Activity *In Vitro* Evaluation

Human PDE4A and PDE4B activities were measured using an IMAP TR-FRET protocol (kit from Molecular Devices, Sunnyvale, CA, USA) according to the instructions of the manufacturer. Briefly, the enzymatic reactions were carried out at room temperature in a 96-well black plate by co-incubating 100 nM FAM-cAMP (R7513), 10 µM putative inhibitory compounds and 4 ng PDE4A or 10 ng PDE4B isoform dissolved in assay buffer (R7364) for 1 h. The enzymes were obtained from human recombinant sources (MDS PHARMA), whereas the other reagents were purchased from Molecular Devices. Fluorescence polarization intensity was measured at 485 nm excitation and 520 nm emission using a microplate reader, SpectraMax M5 (Molecular Devices, Sunnyvale, CA, USA). Roflumilast was dissolved in dimethyl sulfoxide (DMSO) at a final concentration of 0.1%. The vehicle had no significant effect on PDE4 activity in this condition.

### Molecular Dynamics Simulations and Binding-Free Energy Estimations

The simulations were performed using GROMACS simulation version 5 (Van Der Spoel et al., 2005) and CHARMM force field (Vanommeslaeghe et al., 2010). The solvent properties were mimetic using TIP3P water model with a cubic box large enough to allow a minimum of 1.0 nm space from the protein to the box walls. The system charge was neutralized with the addition of ions at the physiological concentration of 0.15 mM. Geometry optimization of the solvated system was performed using the steepest descent algorithm, followed by equilibration simulations with nVT and nPT ensembles keeping the inhibitor and the protein restrained. The temperature was kept at 300°K coupling the system to a V-rescale thermostat (0.1 ps), while the pressure was also kept constant at 1 bar using the Parinello-Rahman coupling algorithm. With the exception of the octahedral configuration structural metals, an unrestrained molecular dynamics simulation was performed until RMSD stabilization. The short range Columbic and Lennard-Jones interaction energies between compound **1** and the surroundings were monitored during the course of the productive simulation step.

To increase the study sample, the ligand binding poses for both isoforms were made based on the previous data found in RCSB PDB Protein Data Bank (Bernstein et al., 1978). The files of all the crystals of PDE4A and PDE4B were used for the alignment between each crystallized ligand and compound **1**. All binding poses resulting from the alignment were filtered to delete the repetitions. To enable the direct comparison between the results of the two isoforms, the exact same poses were used for the molecular dynamics simulations on PDE4A and PDE4B.

After the stabilization of each position, the binding-free energy values were calculated using g_mmpbsa package (Kumari et al., 2014). Between the calculated energy components, the E_MM_ was based on the LJ and Coulomb potential for each complex. The G_polar_ was calculated according to the package and for the G_nonpolar_, the solvent accessible volume (SAV) was chosen as the type of nonpolar solvation model. Considering the results of MM/PBSA, the positions with the most promising binding-free energy were selected to extend the time of simulation, until 14 ns, in order to observe for a longer period the behavior of the complex. The simulations followed the same parameters described above.

## Results and Discussion

### Isolation and Structure Elucidation of Compound 1

Compound **1** was obtained as an amorphous yellow solid and its structure was elucidated by comparison of its observed and reported physical data (Muzitano et al., 2006). ^1^H NMR, ^13^C NMR, HSQC, and HMBC spectra are available as **Figures S2**–**S5**, in the **Supplementary Material**. The ^1^H NMR spectra showed characteristic aglycone signals between 7.32 and 6.18 ppm. The signals in the region of 6.20–6.40 ppm are related with the hydrogens of ring A of the flavonoid. In relation to ring B, the signals are compatible with an ortho coupling. Thus, the pattern of signals and multiplicity observed are compatible with the flavonoid quercetin (3,5,7,3’,4’-pentahydroxyflavonol). Also, the spectra had demonstrated that the compound is *O*-glycosylated at the position C-3 of the ring C.

In addition, the data of ^1^H and ^13^C NMR spectra suggested the presence of two sugar units. In the ^1^H NMR, the signal at 0.90 and 5.29 ppm suggested the presence of a unit of rhamnose. This sugar unit was confirmed by ^13^C NMR spectra and also by the both two-dimensional spectra. The other unit of sugar is evidenced in the ^1^H NMR spectra at 3.10–4.15 ppm. This signals suggested that the second sugar unit is an arabinose attached to the rhamnose unit. In conclusion, it was possible to confirm the compound **1** as quercetin 3-*O-α-L*-arabinopyranosyl-(1→2)-*O-α-L-*rhamnopyranoside (**Table 1**).

**Table 1:** NMR spectroscopic data (500 MHz, DMSO-*d*_6_) for compound 1.

Position	δ_C_, type	δ_H_, (*J* in Hz)
2	156.56, C	
3	134.44, C	
4	177.92, C	
5	157.14, C	
6	98.88, CH	6.18, s
7	164.48, C	
8	93.84, CH	6.38, s
9	161.40, C	
10	104.02, C	
1’	120.61, C	
2’	115.73, CH	7.32, s
3’	145.38, C	
4’	148.78, C	
5’	115.54, CH	6.87, d (8.25)
6’	121.03, CH	7.25, d (8.30)
1’’	101.05, CH	5.29, s
2’’	80.68, CH	4.00, s
3’’	70.41, CH	*
4’’	72.56, CH	*
5’’	70.41, CH	*
6’’	17.56, CH_3_	0.90, d (6.10)
1’’’	106.50, CH	4.08, d (7.15)
2’’’	71.14, CH	3.12, t (9.50)
3’’’	71.88, CH	3.12, t (9.50)
4’’’	67.90, CH	*
5’’’	65.93, CH_2_	3.21, d (12.0)

*Signs obscured by the water signal present in the DMSO-*d*_6_

### TF Strategies

The filtered structure-based inverse VS results showed that quercetin appears to be more interactive with proteins of *Homo sapiens* (**Table S2**, **Supplementary Material**). In respect to the therapeutic activities, it was notable the large number of antiinflammatory and antiproliferative targets which can be linked to the activities already reported in the literature for this natural compound (Anand David et al., 2016).

Among the antiinflammatory targets indicated by TF of quercetin, phosphodiesterase 4B (PDE4B) stood out due to its reported action (Jin et al., 2012; Wittmann and Helliwell, 2013). This result can be related to some studies that had evaluated the PDE4B inhibitory capacity of secondary metabolites, including quercetin (Ko et al., 2004; Townsend and Emala, 2013). PDE4 blocker activity was also described with inhibitors derivatives from this flavonol (Chan et al., 2008), which makes PDE4B a promising target for the study of molecules such as compound **1**.

According to this fact, the PDE4B (PDB ID: 4MYQ) (Fox et al., 2014) was highlighted in the ranking that was comprised of the five best results of molecular docking simulations with the complete structure of compound **1** (**Table S3**, **Supplementary Material**). The simulation illustrated a good similarity with the crystallized molecule and a favorable position of the flavonoid at the binding site of the enzyme. The interactions demonstrated a potential stable binding, indicating a hydrophobic interaction between PHE 618 and the ring C of quercetin, an indirect interaction with Mg^+2^ through the water molecules and hydrogen bonds with ASN 567 and TYR 405 (**Figure S6A**, Supplementary Material). In addition, the 2D representation, provided by the program LigPlot (Wallace et al., 1995), also pointed hydrogen bonds between the amino acid residues HIS 406 and GLU 476 (**Figure S6B**, **Supplementary Material**).

Interestingly, another PDE enzyme was indicated as a possible target for the interaction with the compound **1** (PDE10A2), reinforcing the potential capacity of PDE inhibition by this flavonoid (**Table S3**, **Supplementary Material**). The PDE10A2 is a unique gene that encodes the PDE10 family and it is expressed mostly in the brain (Keravis and Lugnier, 2012). Because of its restricted distribution, most tests evaluated the use of its inhibitors for the treatment of neurological diseases (Jørgensen et al., 2013). However, a possible antiinflammatory action has been indicated by a recent study that showed the capacity of small inhibitors to decrease the production of nitrite in LPS-simulated cells (García et al., 2017).

In contrast, the PDE4B inhibitors are widely known for their antiinflammatory action. This enzyme is the major cAMP-metabolizer found in cells of the inflammatory and immune system (Spadaccini et al., 2017) and it is differentiated from the other PDE4 isoforms by its high sensitivity towards the inhibitors (Azam and Tripuraneni, 2014). Among the four isoforms of phosphodiesterase 4 (A-D), PDE4B is believed to play a central role in inflammation, being characterized as the predominant subtype in monocytes and neutrophils (Azam and Tripuraneni, 2014; Yang et al., 2017). Notably, PDE4B has been reported as a pivotal target in the treatment of airway inflammatory changes (Li et al., 2018). This activity is also recognized in the use of the extract of *B. pinnatum* (Cruz et al., 2011), reinforcing the interpretation that the antiinflammatory action of the compound **1** may be a result, at least in part, from the blockade of PDE4B.

The other TF methodology used in this work, the ligand-based VS, also pointed out a target that belongs to the PDE family, increasing the evidences of this possible action by the compound **1**. The cocrystallized ligand within the PDE5A1 enzyme (Ligand PDB ID: 7CA) (Wang et al., 2006) had a similarity score of 0.952, being the only result that showed a similarity score value above 0.5. However, although the targets are both phosphodiesterase enzymes, their therapeutic actions are different and as well as their binding site. The result of protein sequence alignment between the PDE4B and PDE5A1 showed an identity of 25.5% and the difference of their active sites was easily noticed (**Figure S7**, **Supplementary Material**). Therefore, it is probable that compound **1** would not have the same inhibition capacity on PDE4B and PDE5A1.

The choice of the most promising target between the results of both VS techniques was made from a compilation of the lower energies derived from the scoring of docking simulations, the most favorable interaction with the target and the main activity of the extract of *B. pinnatum.* These findings add support to the view that the most promising target for further analyses is indeed the PDE4B enzyme.

### PDE4 Activity *In Vitro* Evaluation

With the aim to investigate the PDE4B blocker capacity appropriate for molecular docking simulations, the *in vitro* evaluation was performed. We also tested the effect of compound **1** on PDE4A to determine the selectivity of our compound with the two PDE4 isoforms that are particularly important to the antiinflammatory action regulation (Manning et al., 1999).

The results of compound **1** were considered expressive concerning both isoforms, but the effect upon PDE4B seemed to be superior. It is important to highlight that the inhibition of PDE4B was even better than the standard drug (roflumilast). Furthermore, the results suggest a relative selectiveness between the PDE4A and PDE4B (**Table 2**).

**Table 2:** Results of PDE4 *in vitro* inhibition.

PDE4A	
**Compound (10 µM)**	**% Inhibition**
Roflumilast	100 ± 9,58
Compound **1**	49,8 ± 7,66
PDE4B	
**Compound (10 µM)**	**% Inhibition**
Roflumilast	85,7 ± 0,68
Compound **1**	100 ± 0

Although the PDE4 is widely studied, the application of phosphodiesterase inhibitors in medicine is still compromised by their side effects, which include nausea and emesis, meaning that their use is limited (Spina, 2008). The exact mechanism that explains their side effects is still unclear, but one of the most accepted explanations involves the nonselective inhibitor effects on the four PDE4 isoforms (Wang et al., 2007). The limitation concerning selectivity is easily justified when the structures of the PDE4 isoforms are analyzed. Their structures differ only in *N*-terminal regions, reflecting the extreme conservation of amino acid residues in their activity sites, particularly between PDE4B and PDE4D (Cheung et al., 2007). In this way, the results showed this flavonoid to be not just a potent PDE4B inhibitor but also a promising compound for the design of other selective analogues.

### Molecular Dynamics Simulations and Binding-Free Energy Estimations

The close similarity between the binding sites of the two isoform makes the elucidation of the selectivity a challenging task. Therefore, there are some studies in literature that used experimental tests trying to identify crucial differences between these isoforms (Wang et al., 2007; Feng et al., 2018). However, the reasons behind the PDE4 subfamily selective inhibition are still unclear when it is based essentially on the binding sites of these enzymes. In this context, the use of alternative methods, such as *in silico* studies, can be a promising way to discover different findings and hypotheses related to this issue. Between them, the docking simulations, used in the VS study, have a particular disadvantage due to their lock-key assumption (Mezei, 2003; Andrusier et al., 2008). In contrast, the molecular dynamics simulation (MD) can theoretically predict the move of every atom contained in the system over time, capturing important findings related to the binding process (Hollingsworth and Dror, 2018). Because of this, MD was considered the most promising method to come to a hypothesis that would attempt to explain the selective inhibition demonstrated by compound **1** and, consequently, assist in the design of new selective PDE4B inhibitors.

**Figure 2:**
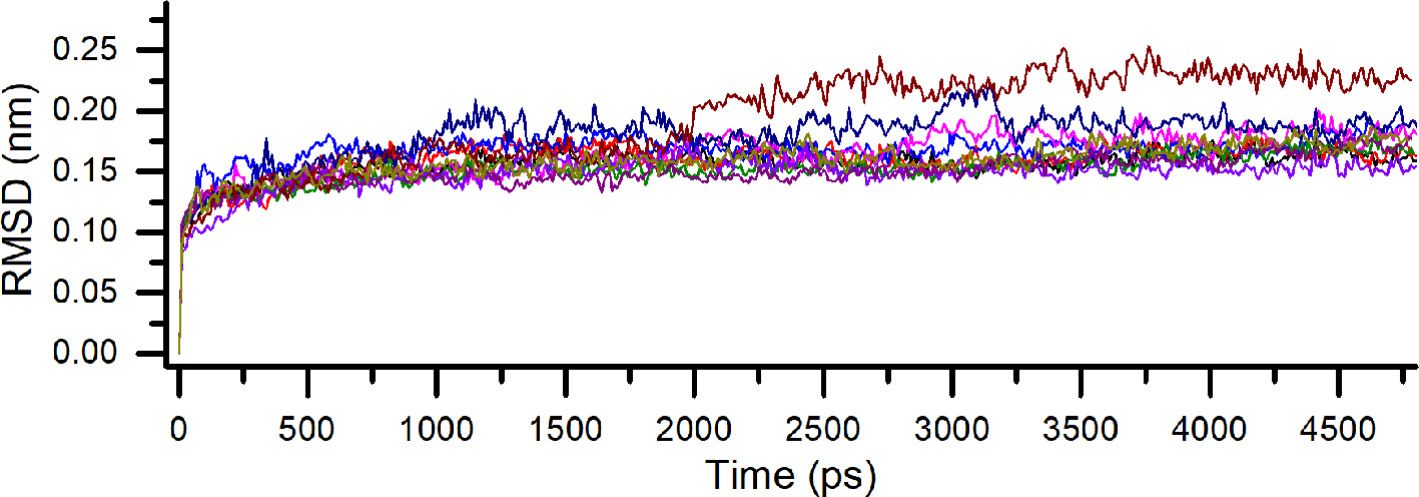
Comparative root-mean-square deviation (RMSD) for all binding poses of compound 1 on PDE4A and PDE4B. Each pose is indicated by a different color.

One of the most widely used parameters in MD simulations is the root-mean-square deviation (RMSD). The RMSD reflects the average displacement of the atoms and its use allows us to determine the structural stabilization in the time scale of the simulation period (Martínez, 2015; Sargsyan et al., 2017). Every one of the five possible positions of the flavonoid was submitted to a lower simulation time before the further analyzes aiming to work with already optimized complexes. The RMSD of each binding pose of the flavonoid on PDE4A (PDB ID: 2QYK) (Wang et al., 2007) and PDE4B (PDB ID: 3O57) (Mitchell et al., 2010) demonstrated a complete optimization of the protein backbone before 5.0 ns, without a significant change of conformation by this time. This fact indicates that the time of the simulations was enough for the refinement of all the structures in the period used. In addition, the high similarity of the RMSD profile of each pose on both isoforms indicates another reflection of their resemblance (**Figure 2**).

To determine the better positions of compound **1** at the activity sites of both isoforms, a Molecular mechanics Poisson-Boltzmann surface area method (MM/PBSA) was carried out. This approach has been used in a lot of studies and can be combined with MD simulations to give important entropic contributions to the total binding energy (Homeyer and Gohlke, 2012; Kumari et al., 2014; Genheden and Ryde, 2015). It is important to emphasize the influence of the choice of nonpolar models in the binding-free energy results. Among the models, the solvent accessible surface area (SASA) is one of the most widely implemented nonpolar types of modeling in the MM/PBSA (Kumari et al., 2014). However, previous works have shown the difficulties of this model in the discrimination of conformational states in explicit solvent simulations, producing bias (Wagoner and Baker, 2006). To overcome this issue, other nonpolar solvent models were tested, such as the SAV and the hybrid model (SASA-SAV) (Wagoner and Baker, 2006; Kumari et al., 2014). In contrast with the SASA-only model, the data results showed that the SAV-only model can reproduce the explicit solvent nonpolar simulation with promising fidelity (Wagoner and Baker, 2006). Therefore, the SAV-only model was chosen for our analysis. Additionally, in MM/PBSA, the result of binding-free energy also takes into account the values of electrostatic, Van der Walls and polar energies (Kumari et al., 2014). These values were computed for every one of the five binding poses for **1** on the two isoforms (**Table 3**).

**Table 3:** Calculated values that contributed to binding-free energy from MM/PBSA^a^.

	Pose	ΔE_vdw_	ΔE_elec_	ΔG_polar_	ΔG_SAV_	ΔG_bind_
**PDE4A**	**1**	−51.73 ± 0.34	−8.20 ± 0.21	34.64 ± 0.39	−39.10 ± 0.55	−64.42 ± 0.65
	**2**	−53.73 ± 0.58	0.14 ± 0.35	61.21 ± 2.0	−38.52 ± 1.03	−30.86 ± 2.29
	**3**	−45.93 ± 0.53	−11.52 ± 0.43	63.60 ± 3.52	−39.23 ± 1.19	−32.96 ± 2.59
	**4**	−46.42 ± 0.53	−11.09 ± 0.49	35.08 ± 0.72	−34.59 ± 0.82	−57.07 ± 1.01
	**5**	−41.88 ± 0.15	−7.20 ± 0.14	60.30 ± 0.86	−31.35 ± 0.22	−20.12 ± 0.86
**PDE4B**	**1**	−65.80 ± 0.44	−25.98 ± 0.43	73.587 ± 0.49	−54.40 ± 0.52	−72.58 ± 0.77
	**2**	−48.77 ± 0.63	−8.23 ± 0.34	37.30 ± 0.41	−35.84 ± 0.84	−55.60 ± 1.19
	**3**	−54.53 ± 0.67	1.18 ± 0.27	51.62 ± 0.70	−40.05 ± 1.02	−41.84 ± 1.41
	**4**	−54.40 ± 0.43	−6.44 ± 0.35	53.79 ± 0.79	−45.27 ± 1.09	−52.27 ± 1.30
	**5**	−56.63 ± 0.22	−21.89 ± 0.25	54.07 ± 0.28	−47.94 ± 0.25	−72.38 ± 0.35

*a:* All values, standard deviations and averages, are expressed in kcal mol^-1^.

The results demonstrated that, in the vast majority of the poses, the flavonoid had a considerably better binding-free energy value on the PDE4B isoform. These values reinforce the hypothesis of a more favorable interaction in a molecular scenario between the compound **1** and PDE4B and also corroborates with the *in vitro* results. Among the binding poses, the first and fifth ones stood out. The first pose, in particular, had the most promising binding-free energy result on the both PDE4A and PDE4B enzymes (−64.42 and −72.58 kcal mol^-1^, respectively) which shows it to be an interesting binding position at the two activity sites. The fifth pose had a very similar binding energy on PDE4B to the first one (−72.38 kcal mol^-1^) and can be considered as a potential occupation mode at the binding site of PDE4B as well.

**Figure 3:**
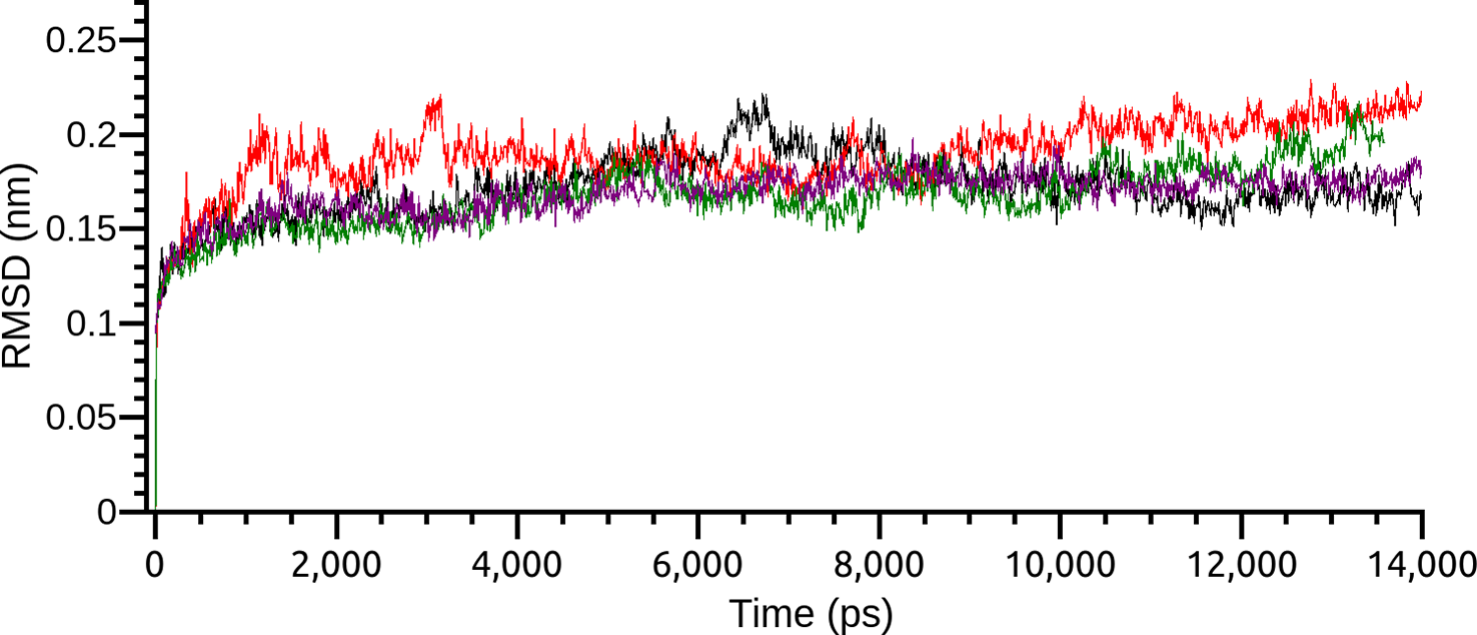
Root-mean-square deviation (RMSD) of the two most interesting binding poses of compound 1 on PDE4A and PDE4B. Each pose is indicated by a different color.

Based on the results of MM/PBSA, the two most promising positions were selected to extend the time of MD simulations in order to analyze the complex behavior for a longer period. The RMSD of the two different poses, as before, demonstrated a very stable profile of optimization with a complete stabilization before 5.0 ns. Both pose conformations on PDE4A and PDE4B remained stable after the extension of the simulation time (**Figure 3**).

The output results after the extension of the MD time were also analyzed based on the relevant features of the binding site of each isoform. The structure of the activity site and the binding position of cAMP in the PDE4 enzyme are essential to the evaluation of the inhibitor action. The activity of PDE4 enzyme depends on the recognition of the cAMP’s nucleotide followed by its hydrolysis. Structurally, the x-ray crystallography of the PDE4 indicated the three most relevant pockets for the activity of this enzyme (Spina, 2008). At the Q pocket, the cAMP is identified by the formation of hydrogen bonds with the nucleotide adenosine and, once it is recognized, the phosphate moiety complex with the metals (Zn^2+^, Mg^2+^) of the enzyme’s bivalent metal-binding pocket (M pocket) promoting its hydrolysis (Houslay et al., 2005; Spina, 2008). The third pocket (S pocket) has polar amino acid residues and an interesting contact with the external solvent, probably contributing to the stabilization of the inhibitor at the binding site. In this context, the majority of PDE4 inhibitors show a structure capable of occupying specifically the Q and M pockets to prevent the entry and hydrolysis of cAMP (Houslay et al., 2005).

**Figure 4:**
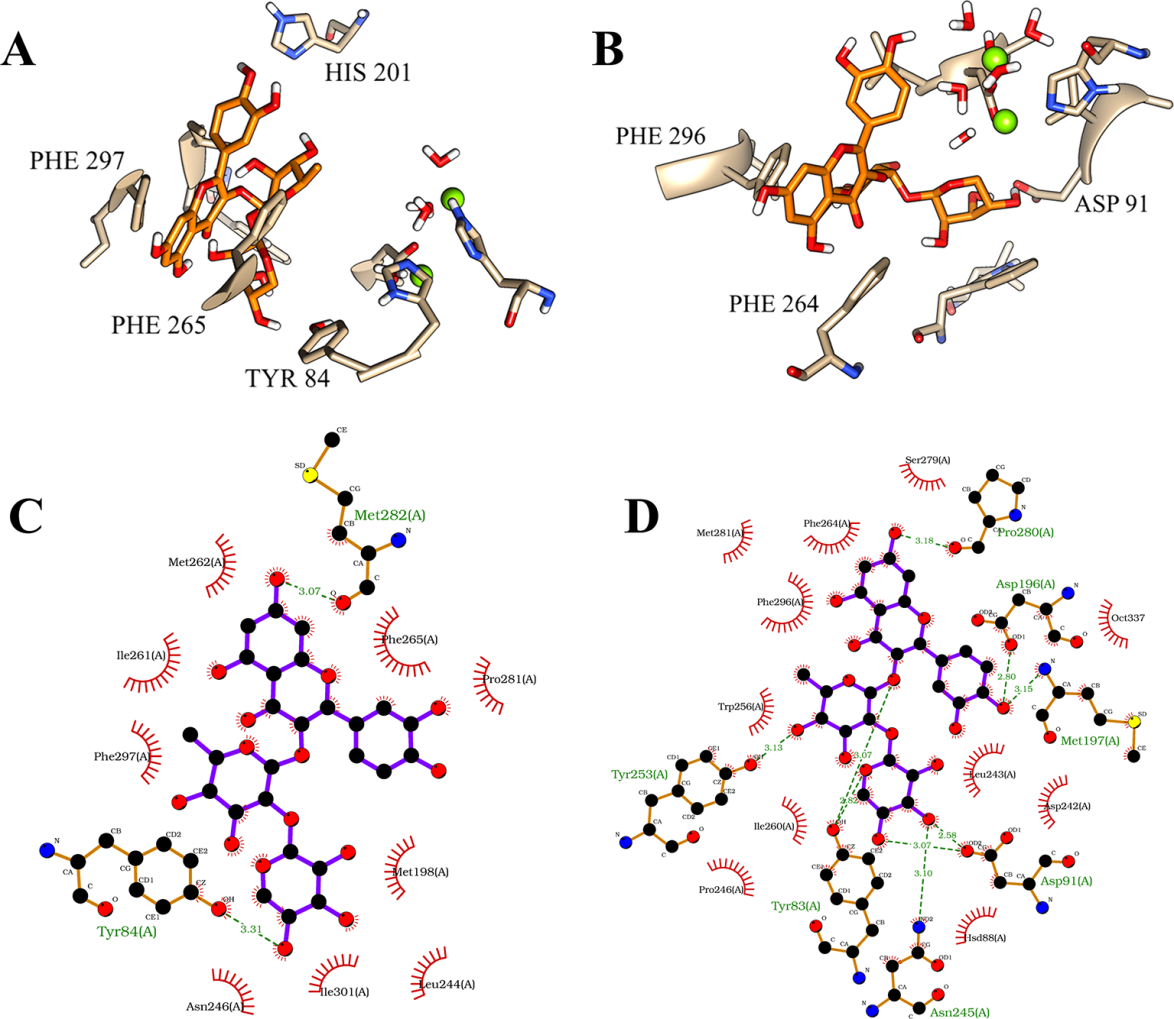
Illustration of interactions between compound 1 (orange) and the amino acid residues of PDE4A **(A)** and PDE4B **(B)** for the first pose. 2D representation of the intermolecular interactions between the compound 1 and PDE4A **(C)** and PDE4B **(D)**. The 2D diagrams was provided by the program LigPlot.

Considering this structural information, the first position of the flavonoid demonstrated considerable intermolecular differences between its occupations at both of the binding sites of the isoforms. On PDE4A, the compound **1** showed a very stable position at the Q pocket with a double parallel π-stacking interaction between the amino acid residues PHE 296 and PHE 265, and also the hydrogen bonds with the residues HIS 201 and TYR 84. However, the compound clearly had an important dislocation at the activity site and had no interactions with the metals of the M pocket (**Figure 4A**). As already mentioned, the interactions with the bivalent metals influence the inhibition of PDE4 enzymes in a direct way (Houslay et al., 2005). Therefore, the behavior of the best pose of the flavonoid indicated by MM/PBSA on PDE4A represents a valid hypothesis for the lower inhibition capacity of compound **1** on this isoform. In contrast, the compound **1** at the activity site of PDE4B also demonstrated the occupation of the Q pocket, showing one parallel π-stacking interaction with the amino acid residue PHE 296. Additionally, there was an increase of hydrogen bonds between the glycosides and the polar amino acid residues and an indirect interaction with the metals through hydrogen bonds with the water molecules (**Figure 4B**). This increase was clearly demonstrated in the 2D representation of the intermolecular interactions of compound **1** at both binding sites. It was detected the presence of 8 valid hydrogen bonds between the natural product and the amino acid residues of PDE4B (**Figure 4D**) and only 2 valid hydrogen bonds with the amino acid residues of PDE4A (**Figure 4C**).

**Figure 5:**
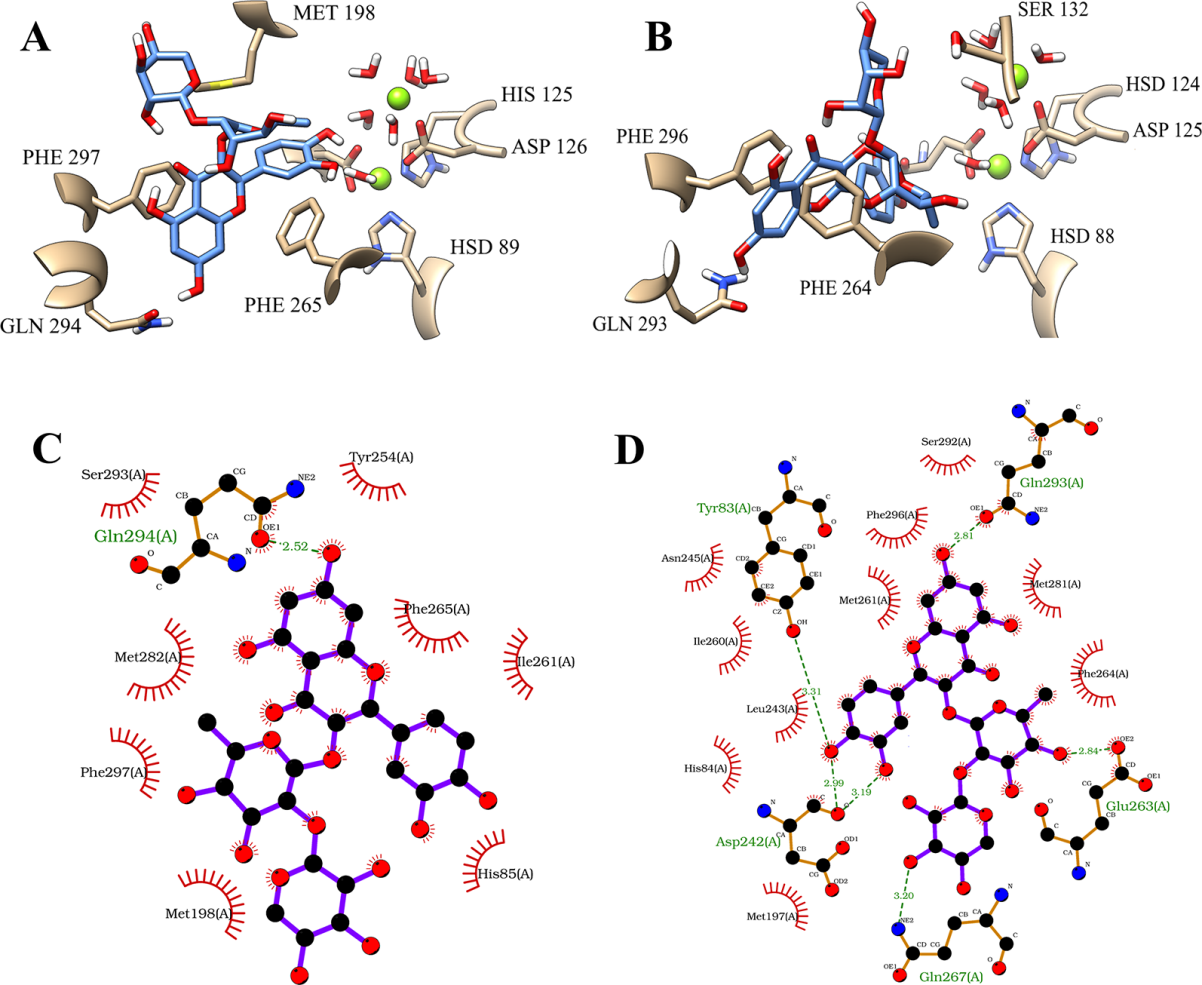
Illustration of interactions between compound 1 (blue) and the amino acid residues of PDE4A **(A)** and PDE4B **(B)** for the fifth pose. 2D representation of the intermolecular interactions between the compound 1 and PDE4A **(C)** and PDE4B **(D)**. The 2D diagrams was provided by the program LigPlot.

The fifth pose also demonstrated a promising result. This binding position showed a large difference of the binding-free energy of the flavonoid on PDE4A and PDE4B. However, in particular, the results of MM/PBSA on PDE4B pointed to a very similar binding-free energy in comparison with the first pose. Interestingly, the analyses on both the binding sites demonstrated that the most significant variation involved the hydrophobic interactions with the amino acid residues of phenylalanine present in the Q pocket. At the PDE4A, the flavonoid exhibited a parallel π-stacking interaction with PHE 297 (**Figure 5A**). In comparison, the compound **1** had a double parallel π-stacking with PHE 296 and PHE 264 at the PDE4B (**Figure 5B**). However, even with these similar interactions, the results of binding-free energy clearly indicate disadvantages of this pose at the PDE4A activity site. Analyzing the 2D representations, it was possible to confirm the higher number of hydrogen bonds between the compound **1** and the amino acid residues of PDE4B (**Figure 5D**) in comparison with PDE4A (**Figure 5C**). This difference may be related with the better interaction with PDE4B pointed by the favorable binding-free energy value.

Another interesting fact involving these two potential binding poses is the positional reflection between them. However, the behaviors of the poses on PDE4A and PDE4B were substantially different. In regard to the PDE4B isoform, both poses were stable at the activity site. In the first pose the glycosides occupied the polar region between the Q and M pockets and the fifth pose demonstrated that the compound **1** was also exploring polar interactions but at the hydrated S pocket. The same was not observed in PDE4A. The difference in the binding-free energy results of the first and fifth poses (−64.42 and −20.12 kcal mol^-1^, respectively) was reflected in the interaction of these binding positions in the two isoforms. The compilation results on PDE4A, demonstrated that even the first pose which had the most promising binding-free energy, showed major hindrances in the occupation of the activity site.

The traditional binding pose observed for the known PDE4 inhibitors, including the Roflumilast, had demonstrated that the most important occupation at the active site is with the amino acids belonging to the Q and M pockets (**Figure S8**, Supplementary Material). Especially three types of interaction are crucial to the PDE4 inhibition: interactions with the metal ions through structural water molecules, hydrogen bonds and hydrophobic interactions with the amino acid residues at Q pocket (Card et al., 2004). In this molecular scenario, compound **1** followed the traditional binding pose exhibited by most of the PDE4 inhibitors and had a widespread occupation of the activity site. However, the occupation in PDE4B was demonstrated to be higher after the molecular dynamics simulations, suggesting a formation of a stable system of hydrogen bonds and hydrophobic interactions. Therefore, the small but significative differences between the PDE4 subfamilies found in previous literature on experimental tests (Wang et al., 2007; Feng et al., 2018), seems to be crucial to the whole complex behavior simulated *in silico*.

Although compound **1** showed interesting *in vitro* and *in silico* results, it is important to emphasizing that this compound has disadvantages as a drug candidate. The enzyme lactase phrorizin hydrolase, found in small intestine of mammalians, is capable of hydrolyzing a range of flavonol and isoflavone glycosides which difficult the abortion of these compounds as a whole molecule (Day et al., 2000). Since the molecular dynamics simulations had demonstrated the importance of the glyosidic portion for the PDE4 blocker action and selectivity profile, potential medications consisting only of compound **1** would not be optimal for per oral, the most common type of administration. However, it can be considered as a promising lead compound and the results of molecular dynamics simulations may be used to design novel potential PDE4B inhibitors based on the structure of compound **1**. Aiming to increase the synthetic accessibility of the designed compounds, the glycosides may be replaced by another polar structure in order to preserve the hydrogen bonds made in S pocket. In contrast, quercetin aglycone appears to be essential for the inhibition of the both isoforms and its replacement should be made by other structure with high similarity.

## Conclusion

Our studies showed that the employed TF methodologies were efficient approaches to identify the alleged activities of natural products, abruptly reducing the costs and numbers of biological models. Based on the results, this approach proved to be an interesting tool for the chemical and pharmacological investigation of possible natural extract markers such as compound **1**. The flavonoid quercetin 3-*O-α-L*-arabinopyranosyl-(1→2)-*O-α-L-*rhamnopyranoside, one of the major compounds of *B. pinnatum*, had its antiinflammatory activity explained by the inhibition of PDE4B. This action confirmed by *in vitro* evaluation, indicates that compound **1** is not just a potent PDE4 blocker, but also demonstrated it to be highly selective to PDE4B. In addition, it was possible to explore this inhibiting and selectivity action of compound **1** with molecular dynamics simulations which shed light on the atomic level inhibition properties of this compound. These results show important progress in the investigation of the antiinflammatory properties of *B. pinnatum* and reinforced the possible choice of compound **1** as the extract chemical marker of this plant. Finally, these highly expressive results show this flavonoid to be an interesting prototype for the design of other PDE4B selective inhibitors.

## Data Availability Statement

All datasets generated for this study are included in the article/**Supplementary Material**.

## Author Contributions

EL, SZ, and EB conceived and designed the experimental tests. The development of the methodology was made by EL, JF, SZ, VC, MM, and EB. Analysis of the data was made by EL, AJ, VC, MM, and EB. Contributed reagents/materials/analysis: JM, SM, VC, and MM. The paper was written and reviewed by EL, AJ, VC, MM, SZ, and EB.

## Funding

This study was financed in part by the Coordenação de Aperfeiçoamento de Pessoal de Nível Superior - Brasil (CAPES) - Finance Code 001.

## Conflict of Interest

The authors declare that the research was conducted in the absence of any commercial or financial relationships that could be construed as a potential conflict of interest.

## References

[B1] Anand David A. Arulmoli R. Parasuraman S. (2016). Overviews of biological importance of quercetin: a bioactive flavonoid. Pharmacogn. Rev. 10 (20), 84–89. 10.4103/0973-7847.194044 28082789 PMC5214562

[B2] Andrusier N. Mashiach E. Nussinov R. Wolfson H. J. (2008). Principles of flexible protein-protein docking. Proteins 73 (2), 271–289. 10.1002/prot.22170 18655061 PMC2574623

[B3] Azam M. A. Tripuraneni N. S. (2014). Selective phosphodiesterase 4B inhibitors: a review. Sci. Pharm. 82 (3), 453–482. 10.3797/scipharm.1404-08 25853062 PMC4318138

[B4] Barreiro E. J. Bolzani V. D. S. (2009). Biodiversidade: fonte potencial para a descoberta de fármacos. Quim. Nova 679–688. 10.1590/S0100-40422009000300012

[B5] Bernstein F. C. Koetzle T. F. Williams G. J. B. Meyer E. F. Brice M. D. Rodgers J. R. (1978). The protein data bank: a computer-based archival file for macromolecular structures. Arch. Biochem. Biophys. 80 (2), 584–591. 10.1016/0003-9861(78)90204-7 626512

[B6] Card G. L. England B. P. Suzuki Y. Fong D. Powell B. Lee B. (2004). Structural basis for the activity of drugs that inhibit phosphodiesterases. Structure 12 (12) 2233–2247. 10.1016/j.str.2004.10.004 15576036

[B7] Chan A. L. Huang H. L. Chien H. C. Chen C. M. Lin C. N. Ko W. C. (2008). Inhibitory effects of quercetin derivatives on phosphodiesterase isozymes and high-affinity [3H]-rolipram binding in guinea pig tissues. Invest. New Drugs 26 (5), 417–424. 10.1007/s10637-008-9114-7 18264679

[B8] Cheung Y.-F. Kan Z. Garrett-Engele P. Gall I. Murdoch H. Baillie G. S. (2007). PDE4B5, a novel, super-short, brain-specific cAMP phosphodiesterase-4 variant whose isoform-specifying N-terminal region is identical to that of cAMP phosphodiesterase-4D6 (PDE4D6). J. Pharmacol. Exp. Ther. 322 (2), 600–609. 10.1124/jpet.107.122218 17519386

[B9] Cruz E. A. Reuter S. Martin H. Dehzad N. Muzitano M. F. Costa S. S. (2011). Kalanchoe pinnata inhibits mast cell activation and prevents allergic airway disease. Phytomedicine 19 (2), 115–121. 10.1016/j.phymed.2011.06.030 21802918

[B10] Day A. J. Cañada F. J. Díaz J. C. Kroon P. A. McLauchlan R. Faulds C. B. (2000). Dietary flavonoid and isoflavone glycosides are hydrolysed by the lactase site of lactase phlorizin hydrolase. FEBS Lett. 468 (2–3), 166–170. 10.1016/S0014-5793(00)01211-4 10692580

[B11] dos Santos Nascimento L. B. de Aguiar P. F. Leal-Costa M. V. Coutinho M. A. S. Borsodi M. P. G. Rossi-Bergmann B. (2018). Optimization of aqueous extraction from kalanchoe pinnata leaves to obtain the highest content of an anti-inflammatory flavonoid using a response surface model. Phytochem. Anal. 29 (3), 308–315. 10.1002/pca.2744 29349835

[B12] Espargaró A. Ginex T. Vadell M. del M. Busquets M. A. Estelrich J. (2017). Combined *in vitro* Cell-Based/*in silico* screening of naturally occurring flavonoids and phenolic compounds as potential anti-alzheimer drugs. J. Nat. Prod. 80 (2), 278–289. 10.1021/acs.jnatprod.6b00643 28128562

[B13] Ezuruike U. F. Prieto J. M. (2014). The use of plants in the traditional management of diabetes in nigeria: pharmacological and toxicological considerations. J. Ethnopharmacol. 155 (2), 857–924. 10.1016/j.jep.2014.05.055 24929108

[B14] Feng X. Wang H. Ye M. Xu X. T. Xu Y. Yang W. (2018). Identification of a PDE4-Specific Pocket for the Design of Selective Inhibitors. Biochemistry 57 (30), 4518–4525. 10.1021/acs.biochem.8b00336 29975048 PMC6088244

[B15] Fernandes J. M. Félix-Silva J. da Cunha L. M. Gomes J. A. dos S. Siqueira E. M. S. (2016). inhibitory effects of hydroethanolic leaf extracts of kalanchoe brasiliensis and kalanchoe pinnata (crassulaceae) against local effects induced by bothrops jararaca snake venom. PloS One e0168658. 10.1371/journal.pone.0168658 28033347 PMC5199091

[B16] Fernandes J. M. Cunha L. M. Azevedo E. P. Lourenço E. M. G. Fernandes-Pedrosa M. F. Zucolotto S. M. (2019). Kalanchoe laciniata and Bryophyllum pinnatum: an updated review about ethnopharmacology, phytochemistry, pharmacology and toxicology. Rev. Bras. Farmacogn. 29 (4), 529–558. 10.1016/j.bjp.2019.01.012

[B17] Fox D. Burgin A. B. Gurney M. E. (2014). Structural basis for the design of selective phosphodiesterase 4B inhibitors. Cell. Signal. 26 (3), 657–663. 10.1016/j.cellsig.2013.12.003 24361374 PMC4057648

[B18] Freires I. A. Sardi J. de C. O. de Castro R. D. Rosalen P. L. (2017). Alternative animal and non-animal models for drug discovery and development: bonus or burden? Pharm. Res. 34, 681–686. 10.1007/s11095-016-2069-z 27858217

[B19] García A. M. Brea J. González-García A. Pérez C. Cadavid M. I. Loza M. I. (2017). Targeting PDE10A GAF domain with small molecules: A way for allosteric modulation with anti-inflammatory effects. Molecules 22 (9), E1472. 10.3390/molecules22091472 28869560 PMC6151459

[B20] Genheden S. Ryde U. (2015). The MM/PBSA and MM/GBSA methods to estimate ligand-binding affinities. Expert Opin. Drug Discovery 10 (5), 449–461. 10.1517/17460441.2015.1032936 PMC4487606 25835573

[B21] Hollingsworth S. A. Dror R. O. (2018). Molecular Dynamics Simulation for All. Neuron 99 (6), 1129–1143. 10.1016/j.neuron.2018.08.011 30236283 PMC6209097

[B22] Homeyer N. Gohlke H. (2012). Free energy calculations by the Molecular Mechanics Poisson-Boltzmann Surface Area method. Mol. Inform. 31 (2), 114–122. 10.1002/minf.201100135 27476956

[B23] Horbal L. Marques F. Nadmid S. Mendes M. V. Luzhetskyy A. (2018). Secondary metabolites overproduction through transcriptional gene cluster refactoring. Metab. Eng. 49, 299–315. 10.1016/j.ymben.2018.09.010 30240601

[B24] Houslay M. D. Schafer P. Zhang K. Y. J. (2005). Keynote review: Phosphodiesterase-4 as a therapeutic target. Drug Discovery Today 10 (12), 1503–1519. 10.1016/S1359-6446(05)03622-6 16257373

[B25] Jørgensen M. Kehler J. Langgård M. Svenstrup N. Tagmose L. (2013). Selective inhibitors of PDE2, PDE9, and PDE10: Modulators of activity of the central nervous system. Annu. Rep. Med. Chem. 48, 37–55. 10.1016/B978-0-12-417150-3.00004-1

[B26] Jin S. L. Ding S. L. Lin S. C. (2012). Phosphodiesterase 4 and its inhibitors in inflammatory diseases. Chang Gung Med. J. 35, 197–210. 22735051 10.4103/2319-4170.106152

[B27] Keravis T. Lugnier C. (2012). Cyclic nucleotide phosphodiesterase (PDE) isozymes as targets of the intracellular signalling network: Benefits of PDE inhibitors in various diseases and perspectives for future therapeutic developments. Br. J. Pharmacol. 166 (5), 1288–1305. 10.1111/j.1476-5381.2011.01729.x PMC3372715 22014080

[B28] Ko W. C. Shih C. M. Lai Y. H. Chen J. H. Huang H. L. (2004). Inhibitory effects of flavonoids on phosphodiesterase isozymes from guinea pig and their structure-activity relationships. Biochem. Pharmacol. 68 (10), 2087–2094. 10.1016/j.bcp.2004.06.030 15476679

[B29] Kumari R. Kumar R. Lynn A. (2014). g_mmpbsa – A GROMACS tool for high-throughput MM-PBSA calculations. J. Chem. Inf. Model. 54 (7), 1951–1962. 10.1021/ci500020m 24850022

[B30] Li H. Zuo J. Tang W. (2018). Phosphodiesterase-4 inhibitors for the treatment of inflammatory diseases. Front. Pharmacol. 9, 1048. 10.3389/fphar.2018.01048 30386231 PMC6199465

[B31] Manning C. D. Burman M. Christensen S. B. Cieslinski L. B. Essayan D. M. Grous M. (1999). Suppression of human inflammatory cell function by subtype-selective PDE4 inhibitors correlates with inhibition of PDE4A and PDE4B. Br. J. Pharmacol., 128 (7), 1393–1398. 10.1038/sj.bjp.0702911 10602317 PMC1571768

[B32] Martínez L. (2015). Automatic identification of mobile and rigid substructures in molecular dynamics simulations and fractional structural fluctuation analysis. PloS One e0119264. 10.1371/journal.pone.0119264 25816325 PMC4376797

[B33] Mezei M. (2003). A new method for mapping macromolecular topography. J. Mol. Graph. Model. 21 (15), 463:472. 10.1016/S1093-3263(02)00203-6 12543141

[B34] Mitchell C. J. Ballantine S. P. Coe D. M. Cook C. M. Delves C. J. Dowle M. D. (2010). Pyrazolopyridines as potent PDE4B inhibitors: 5-Heterocycle SAR. Bioorg. Med. Chem. Lett. 20 (19), 5803–5803. 10.1016/j.bmcl.2010.07.136 20732811

[B35] Muzitano M. F. Tinoco L. W. Guette C. Kaiser C. R. Rossi-Bergmann B. Costa S. S. (2006). The antileishmanial activity assessment of unusual flavonoids from Kalanchoe pinnata. Phytochemistry 67 (18), 2071–2077. 10.1016/j.phytochem.2006.06.027 16930642

[B36] Nascimento L. B. S. Leal-Costa M. V. Coutinho M. A. S. Moreira N. D. S. Lage C. L. S. Barbi N. D. S. (2013). Increased antioxidant activity and changes in phenolic profile of kalanchoe pinnata (Lamarck) persoon (crassulaceae) specimens grown under supplemental blue light. Photochem. Photobiol. 148, 73–81. 10.1111/php.12006 23057576

[B37] Nascimento L. B. D. S. Leal-Costa M. V. Menezes E. A. Lopes V. R. Muzitano M. F. Costa S. S. (2015). Ultraviolet-B radiation effects on phenolic profile and flavonoid content of Kalanchoe pinnata. J. Photochem. Photobiol. B Biol. 73–81. 10.1016/j.jphotobiol.2015.03.011 25900552

[B38] Olson A. J. Trott O. (2010). AutoDock Vina: improving the speed and accuracy of docking with a new scoring function, efficient optimization and multithreading. J. Comput. Chem. 31 (2), 455–461. 10.1002/jcc.21334 19499576 PMC3041641

[B39] Pérez G. M. Salomón L. A. Montero-Cabrera L. A. de la Vega J. M. G. Mascini M. (2016). Integrating sampling techniques and inverse virtual screening: toward the discovery of artificial peptide-based receptors for ligands. Mol. Divers. 421, 438. 10.1007/s11030-015-9648-5 26553204

[B40] Panyaphu K. Van On T. Sirisa-Ard P. Srisa-Nga P. Chansakaow S. Nathakarnkitkul S. (2011). Medicinal plants of the Mien (Yao) in Northern Thailand and their potential value in the primary healthcare of postpartum women. J. Ethnopharmacol. 226–237. 10.1016/j.jep.2011.03.050 21458554

[B41] Patridge E. Gareiss P. Kinch M. S. Hoyer D. (2016). An analysis of FDA-approved drugs: Natural products and their derivatives. Drug Discovery Today 204–207. 10.1016/j.drudis.2015.01.009 25617672

[B42] Rester U. (2008). From virtuality to reality - Virtual screening in lead discovery and lead optimization: a medicinal chemistry perspective. Curr. Opin. Drug Discovery Devel. 559–568. 18600572

[B43] Rodrigues R. P. Mantoani S. P. de Almeida J. R. Pinsetta F. R. Semighini E. P. da Silva V. B. (2012). Estratégias de Traigem Virtual no Planejamento de Fármacos. Rev. Virtual Quim. 739–776. 10.5935/1984-6835.20120055

[B44] Sargsyan K. Grauffel C. Lim C. (2017). How Molecular Size Impacts RMSD Applications in Molecular Dynamics Simulations. J. Chem. Theory Comput. 1518–1524. 10.1021/acs.jctc.7b00028 28267328

[B45] Sobreira F. Hernandes L. S. Vetore-Neto A. Díaz I. E. C. de Santana F. C. Mancini-Filho J. (2017). Gastroprotective activity of the hydroethanolic extract and ethyl acetate fraction from Kalanchoe pinnata (Lam.) Pers. Braz. J. Pharm. Sci. 53 (1), e16027. 10.1590/s2175-97902017000116027

[B46] Spadaccini M. D’Alessio S. Peyrin-Biroulet L. Danese S. (2017). PDE4 inhibition and inflammatory bowel disease: A novel therapeutic avenue. Int. J. Mol. Sci. 18 (6), e1276. 10.3390/ijms18061276 28617319 PMC5486098

[B47] Spina D. (2008). PDE4 inhibitors: Current status. Br. J. Pharmacol. 155 (3), 308–315. 10.1038/bjp.2008.307 18660825 PMC2567892

[B48] Sreekeesoon D. P. Mahomoodally M. F. (2014). Ethnopharmacological analysis of medicinal plants and animals used in the treatment and management of pain in Mauritius. J. Ethnopharmacol. 181–200. 10.1016/j.jep.2014.09.030 25261690

[B49] Townsend E. A. Emala C. W. (2013). Quercetin acutely relaxes airway smooth muscle and potentiates -agonist-induced relaxation via dual phosphodiesterase inhibition of PLC and PDE4. AJP Lung Cell. Mol. Physiol. 305 (5), L396–L403. 10.1152/ajplung.00125.2013 PMC3763034 23873842

[B50] Van Der Spoel D. Lindahl E. Hess B. Groenhof G. Mark A. E. Berendsen H. J. C. (2005). GROMACS: Fast, flexible, and free. J. Comput. Chem. 1701–1718. 10.1002/jcc.20291 16211538

[B51] Vanommeslaeghe K. Hatcher E. Acharya C. Kundu S. Zhong S. Shim J. (2010). CHARMM general force field: A force field for drug-like molecules compatible with the CHARMM all-atom additive biological force fields. J. Comput. Chem. 671–690. 10.1002/jcc.21367 19575467 PMC2888302

[B52] Wadood A. Ahmed N. Shah L. Ahmad A. H. H. Shams S. (2013). *In-silico* drug design: An approach which revolutionarised the drug discovery process. Open Access Drug Des. Deliv. 1–3.

[B53] Wagoner J. A. Baker N. A. (2006). Assessing implicit models for nonpolar mean solvation forces: the importance of dispersion and volume terms. Proc. Natl. Acad. Sci. 103 (22), 8331–8336. 10.1073/pnas.0600118103 16709675 PMC1482494

[B54] Wallace A. C. Laskowski R. A. Thornton J. M. (1995). Ligplot: A program to generate schematic diagrams of protein-ligand interactions. Protein Eng. Des. Sel. 127, 134. 10.1002/jcc.24467 7630882

[B55] Wang H. Liu Y. Huai Q. Cai J. Zoraghi R. Francis S. H. (2006). Multiple conformations of phosphodiesterase-5: Implications for enzyme function and drug development. J. Biol. Chem. 281, 21469–21479. 10.1074/jbc.M512527200 16735511

[B56] Wang H. Peng M.-S. Chen Y. Geng J. Robinson H. Houslay M. D. (2007). Structures of the four subfamilies of phosphodiesterase-4 provide insight into the selectivity of their inhibitors. Biochem. J. 193–201. 10.1042/BJ20070970 17727341 PMC2267353

[B57] Wittmann M. Helliwell P. S. (2013). Phosphodiesterase 4 inhibition in the treatment of psoriasis, psoriatic arthritis and other chronic inflammatory diseases. Dermatol. Ther. (Heidelb). 1–15. 10.1007/s13555-013-0023-0 23888251 PMC3680635

[B58] Xu X. Huang M. Zou X. (2018). Docking-based inverse virtual screening: methods, applications, and challenges. Biophys. Reports 1–16. 10.1007/s41048-017-0045-8 PMC5860130 29577065

[B59] Yang J. X. Hsieh K. C. Chen Y. L. Lee C. K. Conti M. Chuang T. H. (2017). Phosphodiesterase 4B negatively regulates endotoxin-activated interleukin-1 receptor antagonist responses in macrophages. Sci. Rep. 7 46165. 10.1038/srep46165 28383060 PMC5382768

[B60] Zoete V. Daina A. Bovigny C. Michielin O. (2016). SwissSimilarity: a web tool for low to ultra high throughput ligand-based virtual screening. J. Chem. Inf. Model. 1399–1404. 10.1021/acs.jcim.6b00174 27391578

