# Supplementary material for: Identification of a Selective PDE4B Inhibitor From Bryophyllum pinnatum by Target Fishing Study and In Vitro Evaluation of Quercetin 3-O-α-L-Arabinopyranosyl-(1→2)-O-α-L-Rhamnopyranoside
Source: Front Pharmacol. 2020 Jan 22;10:1582. doi: 10.3389/fphar.2019.01582 (PMC6987432; doi:10.3389/fphar.2019.01582)
Supplement: Supplementary file 1 [file DataSheet_1.docx]

**Supplementary Material**

**
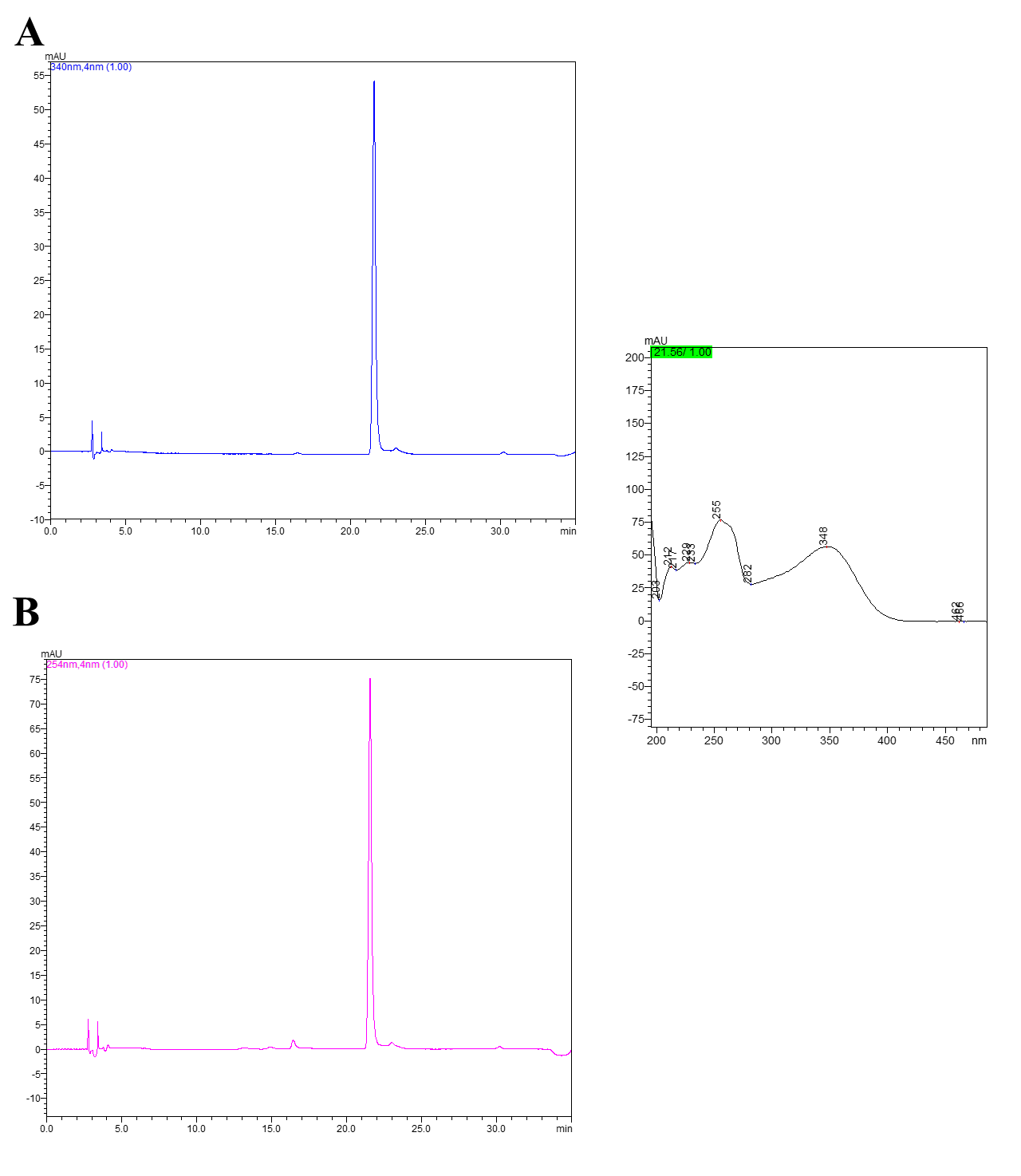
**

**Figure S1**: Chromatogram obtained by UPLC-DAD of compound **1** at 340 and 254nm.


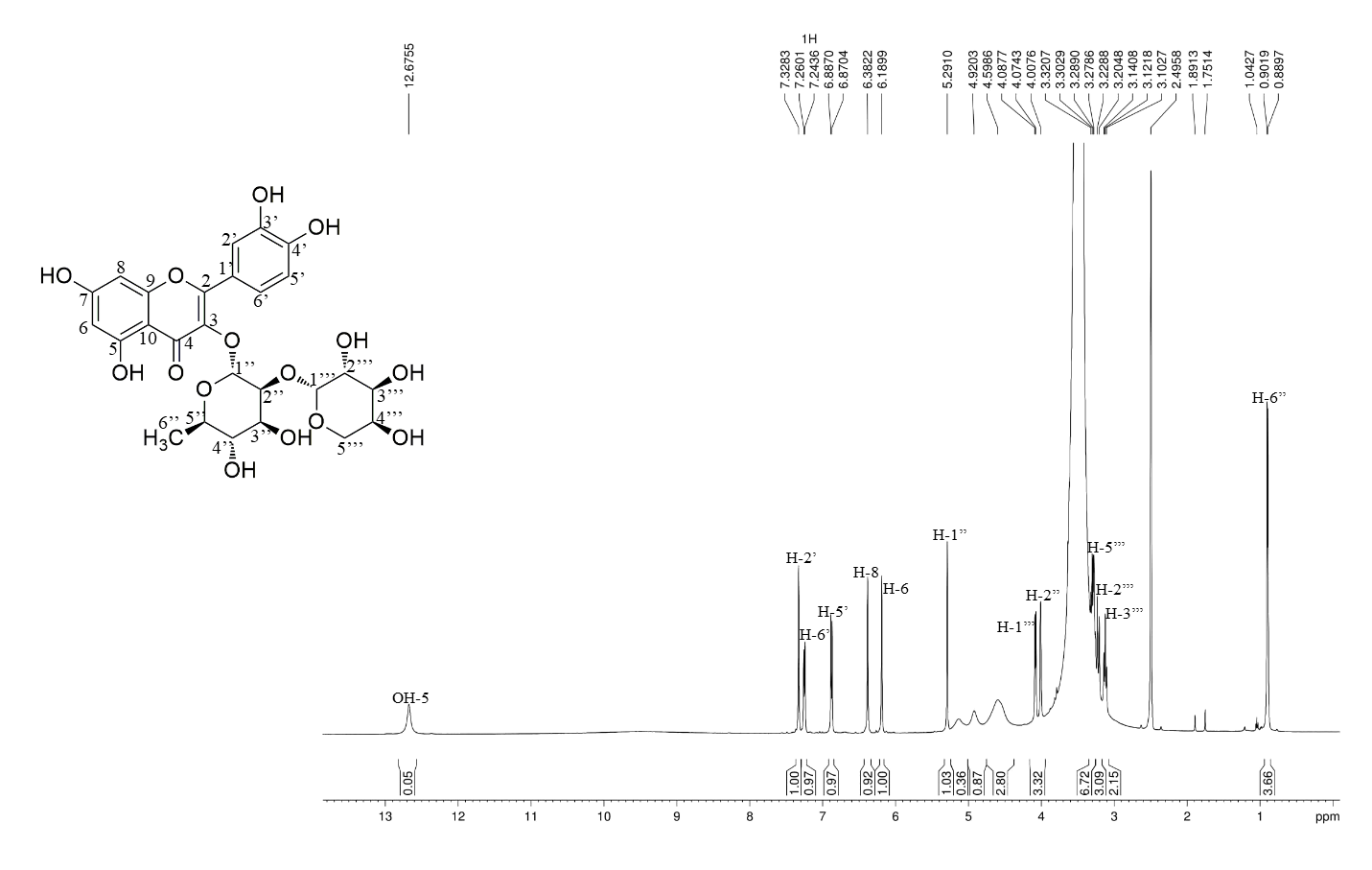


**Figure S2**: ^1^H NMR Spectrum (500 MHz, DMSO-d_6_) of compound **1.**


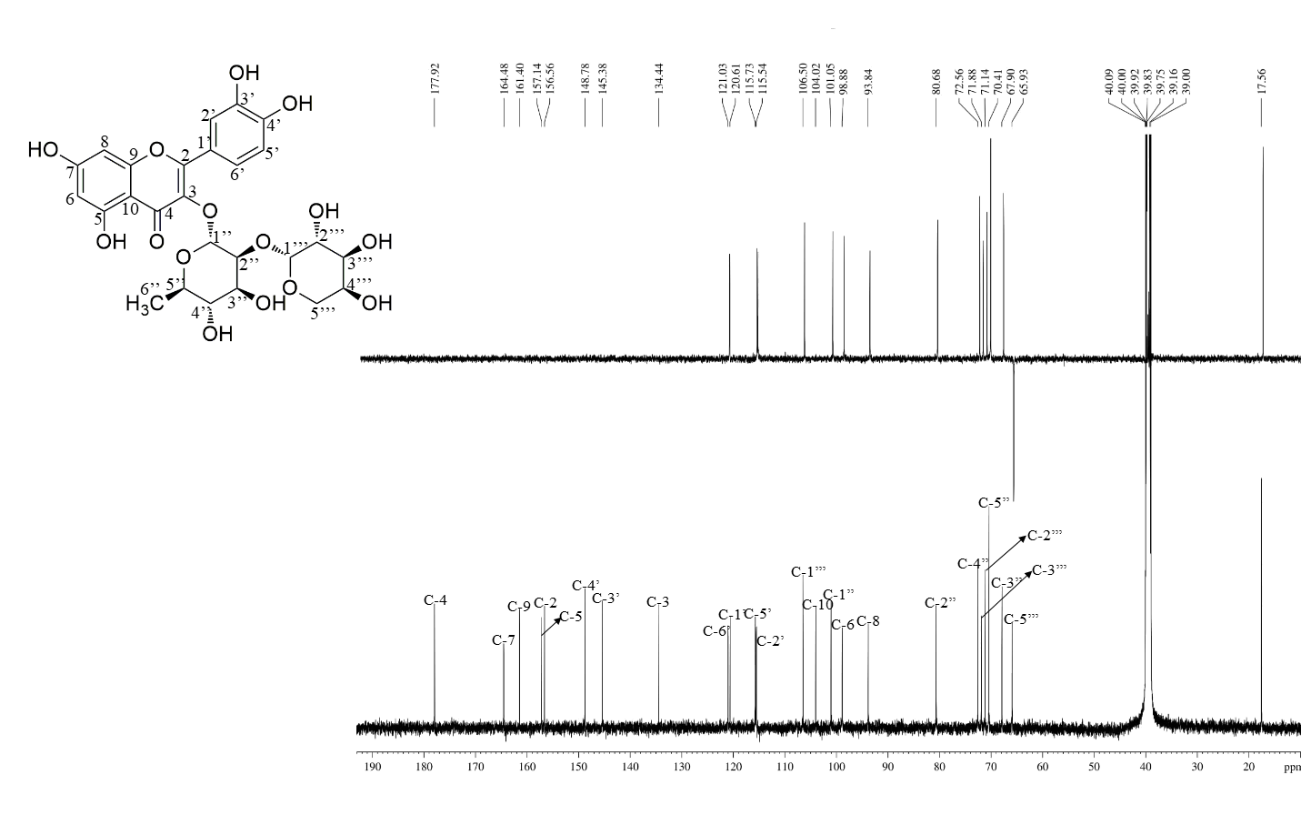


**Figure S3:** ^13^C NMR Spectrum (125 MHz, DMSO-*d*_6_) of compound **1.**

**
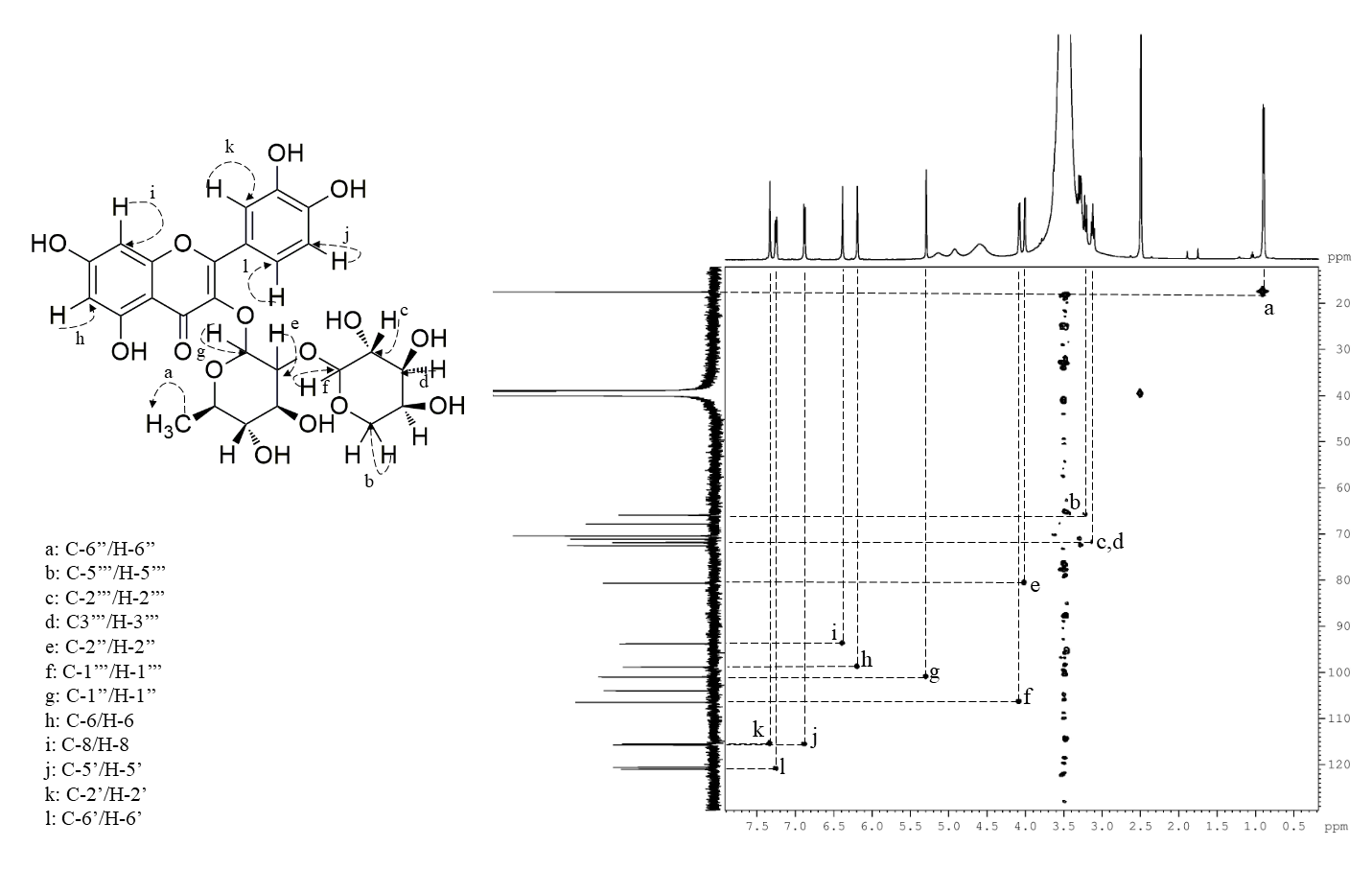
**

**Figure S4:** HSQC Spectrum (125 MHz, DMSO-d_6_) of compound **1.**


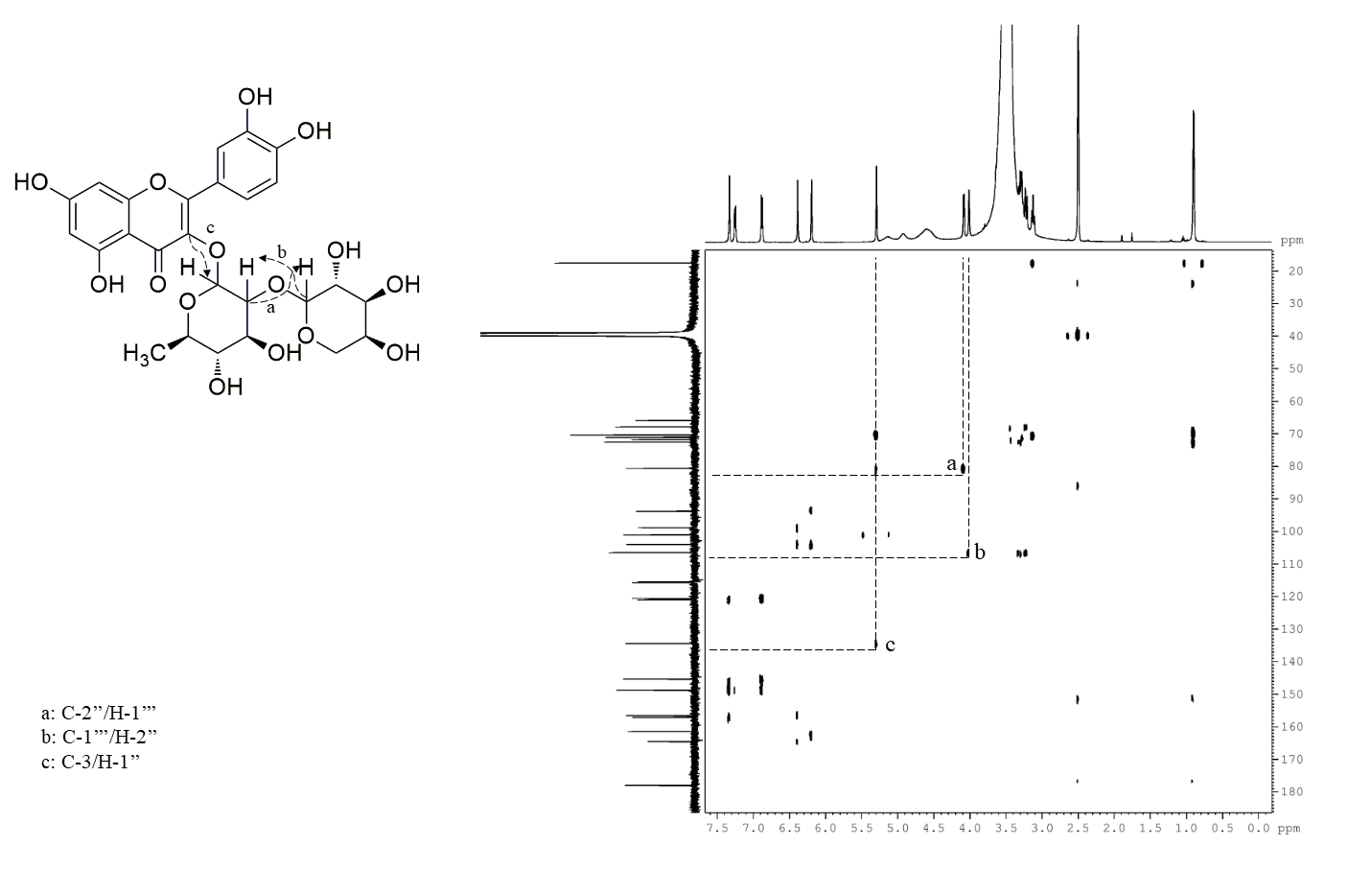


**Figure S5:** HMBC Spectrum (125 MHz, DMSO-d_6_) of compound **1.**

**Table S1:** Chromatographic conditions for the isolation of **1**.

| Column | Analyzed fraction | Chromatographic system | | | |
| --- | --- | --- | --- | --- | --- |
|  |  | Dimensions | Stationary phase | Mobile phase | Flow |
| Column 01 | n-BuOH | 45.7 X 3.7 cm | silica gel 60 | 30% CH_2_Cl_2_: 70-100% AcOEt: 95-0% AcOEt: 5-100% MeOH | Open |
| Colunm 02 | G3 | 40 X 2.0 cm | Sephadex LH-20 | MeOH | 1.0 mL/min |
| Column 03 | G4 | 40 X 2.0 cm | Sephadex LH-20 | MeOH | 1.0 mL/min |

**Table S2:** Ranking with bests the 25 results from Inverse Virtual Screening of quercetin**.**

| **PDB** | **PROTEIN** | **SCORE** |
| --- | --- | --- |
| *4KN2 | Human folate receptor beta (Membrane protein) | -11.2 |
| *4HL5 | Tankyrase 2  (Transferase) | -11.1 |
| *3TE7 | Quinone Oxidoreductase (NQ02)  (oxidoreductase) | -10.8 |
| *2OFV | Lck  (Transferase) | -10.7 |
| **3P2K | Methyltransferase  (Transferase) | -10.5 |
| *3F17 | MMP12 (Hydrolase) | -10.4 |
| ***2Y88 | Phosphoribosyl Isomerase (Isomerase) | -10.4 |
| *4MYQ | Phosphodiesterase 4B  (Hydrolase) | -10.4 |
| *4JPG | PKM2  (Transferase) | -10.3 |
| *1SHJ | Caspase 7  (Hydrolase) | -10.3 |
| *3RIB | Lysine Methyltransferase (Transferase) | -10.3 |
| *2OW9 | MMP13  (Hydrolase) | -10.3 |
| *2JT6 | MMP3  (Hydrolase) | -10.3 |
| *1HY3 | Estrogen Sulfotransferase (Transferase) | -10.3 |
| ****1V0O | PfPK5  (Transferase) | -10.3 |
| *3LDO | GRP78  (Chaperona) | -10.3 |
| *2FPV | DHODH  (Oxidoredutase) | -10.3 |
| *2OVZ | MMP9  (Hydrolase) | -10.3 |
| *2OUQ | PDE10A2  (Hydrolase) | -10.2 |
| **2B82 | AphA class B acid phosphatase/phosphotransferase (Hydrolase) | -10.2 |
| *2FO0 | c-Abl Tyrosine Kinase (Transferase) | -10.2 |
| *1ZXM | Topo IIa  (Isomerase) | -10.2 |
| *2YKI | Hsp90  (Chaperone) | -10.2 |
| *2BP1 | Aflatoxin Aldehyde Reductase (Oxidoreductase) | -10.2 |
| *1PKG | C-Kit Kinase Product ([Transferase Activator](https://www.rcsb.org/pdb/search/smartSubquery.do?smartSearchSubtype=StructureKeywordsQuery&display=true&struct_keywords.pdbx_keywords.value=TRANSFERASE%20ACTIVATOR&struct_keywords.pdbx_keywords.comparator=contains)) | -10.1 |

******Homo sapiens; **Escherichia coli; ****[*Mycobacterium tuberculosis*](https://www.rcsb.org/pdb/search/smartSubquery.do?smartSearchSubtype=TreeEntityQuery&t=1&n=1773)*; *****[*Plasmodium falciparum*](https://www.rcsb.org/pdb/search/smartSubquery.do?smartSearchSubtype=TreeEntityQuery&t=1&n=5833)

**Table S3:** The top five results from Compound A docking simulations.

| **PDB ID** | **PROTEIN** | **SCORE** |
| --- | --- | --- |
| *2OUQ | PDE10A2  (Hydrolase) | -10.3 |
| *2YKI | Hsp90  (Chaperone) | -10.3 |
| *4MYQ | Phosphodiesterase 4B  (Hydrolase) | -10.1 |
| *4EE3 | Human M340H-beta-1,4-galactosyltransferase-1 (Transferase) | -9.8 |
| *2W0D | MMP  (Oxidoreductase) | -9.7 |

*****Homo sapiens

**
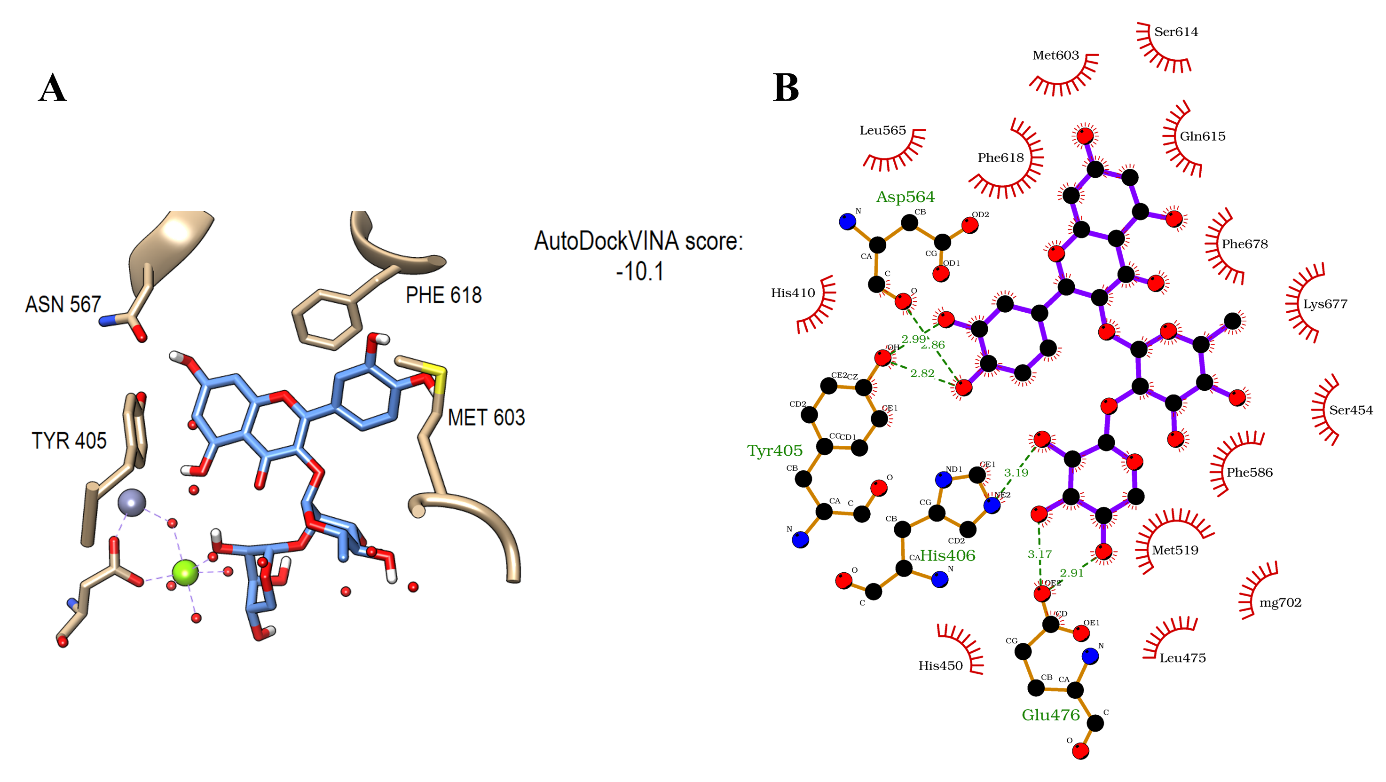
**

**Figure S6:** Illustration of interactions between **1** (blue) and amino acids residues of PDE4B (PDB ID: 4MYQ) in 3D (A) and 2D (B). The 2D diagram was provided by the program LigPlot.


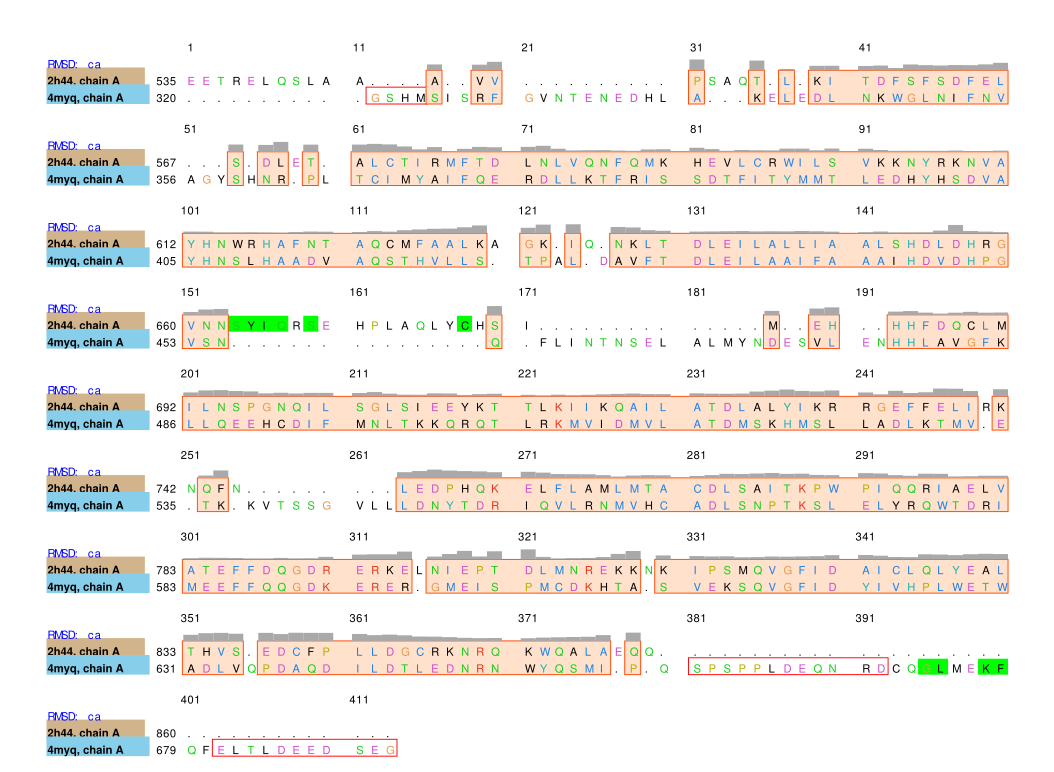


**Figure S7:** Match alignment of PDE5A1 (PDB ID: 2H44) and PDE4B (PDB ID: 4MYQ). The amino acid residues in binding site of both are highlight.


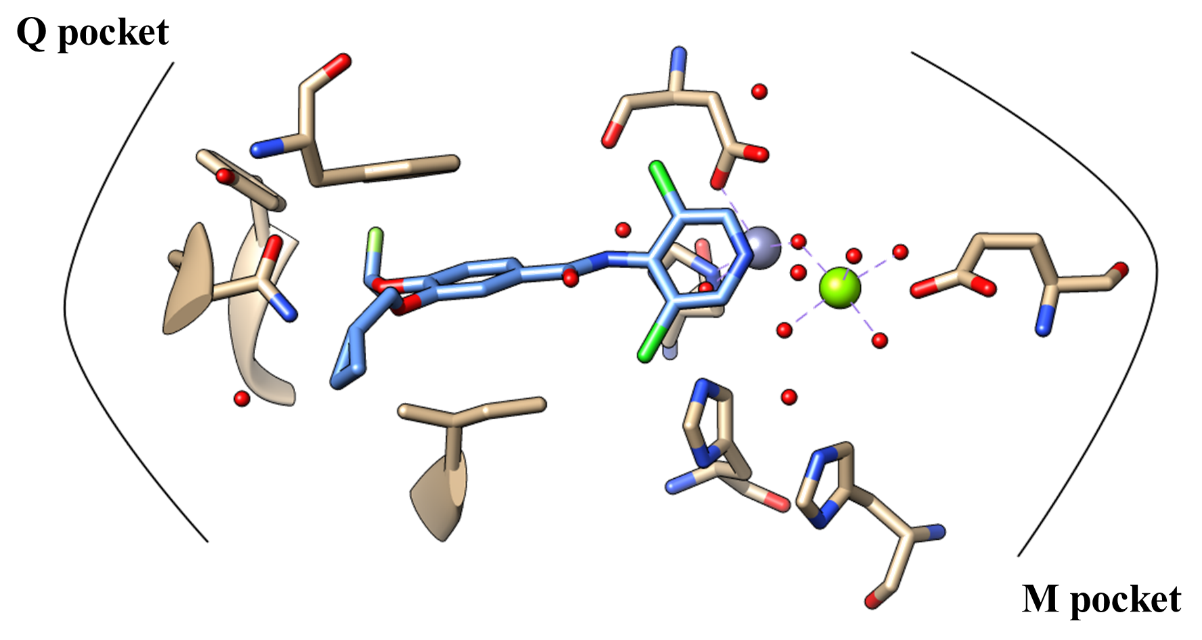


**Figure S8:** Occupation of Roflumilast on PDE4B binding site (PDB ID: 1XMU).
